# Supplementary material for: Prevalence and determinants of anxiety and stress in the general population during COVID-19 pandemic in Iraq: A cross-sectional study
Source: PLoS One. 2023 Apr 12;18(4):e0283260. doi: 10.1371/journal.pone.0283260 (PMC10096275; doi:10.1371/journal.pone.0283260)
Supplement: Attachment — Submitted filename: Plos One Review.docx [file pone.0283260.s001.docx]

This is a well-written manuscript that highlights a topical issue of interest to global health. However, the paper can be improved by considering the under-listed comments.

1. Line 90: What is the total size of the population? How did you arrive at the sample size used?
2. Line 91: The inclusion and exclusion criteria should be clearly stated.
3. Lines: 92 to 96: The statements there should be moved to a section on sampling.
4. Line 96 to 98: From the sentence beginning “The online …. Should be moved to a section on data collection.
5. The authors used an online questionnaire to gather the data. What shows that the intended respondents answered the questionnaire? The limitations of the study should be stated including the weaknesses of using the approach to data collection employed.
6. The discussion section is highly descriptive. The authors can improve upon it by providing explanations as to why they reported a particular result and also explain why a particular support or does not support their study.
7. The paper requires minor editing.
